# Novel Indirect Antioxidant Activity Independent of Nrf2 Exerted by Lactic Acid Bacteria

**DOI:** 10.3390/ijms251910648

**Published:** 2024-10-03

**Authors:** Ayaka Sato, Asami Watanabe, Kyoji Muraki, Hiromi Kimoto-Nira, Makoto Kobayashi

**Affiliations:** 1 Department of Molecular and Developmental Biology, Institute of Medicine, University of Tsukuba, Tsukuba 305-8575, Japan; s2321408@u.tsukuba.ac.jp (A.S.);; 2 Master’s Program in Medical Sciences, Graduate School of Comprehensive Human Sciences, University of Tsukuba, Tsukuba 305-8575, Japan; 3 Institute of Food Research, National Agriculture and Food Research Organization, Tsukuba 305-8642, Japan; anne@affrc.go.jp

**Keywords:** antioxidant activity, H_2_O_2_, Keap1-Nrf2 pathway, lactic acid bacteria, zebrafish

## Abstract

In recent years, the health benefits of lactic acid bacteria have garnered attention, but their antioxidant activity remains relatively underexplored. We have been analyzing the antioxidant activities of various dietary phytochemicals by assessing their ability to mitigate oxidative stressor-induced toxicity in zebrafish larvae through pretreatment. In this study, the antioxidant activities of 24 strains of heat-killed lactic acid bacteria from various origins were examined using this zebrafish assay system. The results revealed that all 24 strains possessed antioxidant activity that reduces hydrogen peroxide toxicity. Further detailed analysis using the H61 strain, which exhibited the strongest activity, showed that no direct antioxidant activity was observed in the assay system, suggesting that the detected antioxidant activity was entirely indirect. Moreover, it was found that pretreatment of zebrafish larvae with the H61 strain for more than 6 h was required to exert its antioxidant activity. This duration was similar to that required by dietary antioxidants that activate the Keap1-Nrf2 pathway, suggesting potential involvement of this pathway. However, analysis using Nrf2-knockout zebrafish revealed that the antioxidant activity of strain H61 is independent of Nrf2, indicating that it represents a novel indirect antioxidant activity that does not involve the Keap1-Nrf2 pathway. To further characterize this activity, the ability to mitigate the toxicity of oxidative stressors other than hydrogen peroxide was examined. The results indicated that while the toxicity of *tert*-butyl hydroperoxide was reduced, unlike with the Keap1-Nrf2 pathway, it was not effective in counteracting the toxicity of paraquat or arsenite, which generate superoxide radicals. In conclusion, we have identified a novel indirect antioxidant activity in lactic acid bacteria.

## 1. Introduction

In a world striving for extended healthy lifespans, dietary antioxidants, both direct and indirect types, are increasingly gaining attention [1,2]. Indirect antioxidants act through the augmentation of cellular antioxidant capacity by enhancing gene expression, such as the kelch-like ECH-associated protein 1 (Keap1)-nuclear factor erythroid-2-related factor 2 (Nrf2) pathway [3]. The function and mechanism of the Keap1-Nrf2 pathway have been studied using zebrafish [4,5,6,7], and it has been found that zebrafish larvae are useful for analyzing and evaluating the indirect antioxidants [8,9]. Using a zebrafish assay system, the indirect antioxidant activities of spice- and soy-derived phytochemicals have been analyzed, revealing seven phytochemicals that exhibit antioxidant effects in zebrafish larvae, with five of these phytochemicals being shown to have Nrf2-dependent antioxidant activities [10,11]. Surprisingly, the antioxidant activities of cinnamon-derived cinnamaldehyde and soy-derived equol were Nrf2-independent. This discovery suggested the potential existence of novel biological pathways that, independently or in conjunction with the Keap1-Nrf2 pathway, exert antioxidant effects within the animal body. Therefore, various food ingredients were analyzed with the hypothesis that other dietary antioxidants with Nrf2-independent indirect antioxidant activities should be present.

Lactic acid bacteria (LAB) are a group of Gram-positive bacteria that include genera such as *Lactobacillus*, *Leuconostoc*, *Pediococcus*, *Lactococcus*, *Enterococcus*, and *Streptococcus*, commonly found in fermented dairy foods [12,13]. As probiotics, live LAB offer various human health benefits, such as balancing and restoring the intestinal microbiota, protecting against pathogens, immunomodulation, and maintaining intestinal barrier integrity. One of the most important aspects of LAB application is a functional evaluation of probiotics using animal models like rats, mice, pigs, chickens, and zebrafish [14]. However, because zebrafish are aquatic and grow at 28 °C with gut microbiota different from humans, their use in human applications has been limited. Recently, interest in heat-killed LAB has increased, as research has shown that the beneficial effects of probiotics are not necessarily dependent on live bacteria [15,16,17]. Heat-killed LAB have demonstrated the ability to restore normal intestinal homeostasis and exert immunomodulatory and pathogen-antagonistic effects similar to those of live LAB. The use of heat-killed LAB would mitigate safety concerns related to live microorganisms and the minimal regulation surrounding probiotics. Furthermore, since the LAB are no longer living, differences in growth temperature and gut microbiota between zebrafish and humans would no longer be a significant concern. Zebrafish, especially their embryos and larvae, are small and prolific, and they develop quickly in vitro with minimal ethical concerns, making them advantageous over other vertebrates in terms of time, cost, and space for chemical compound analyses. However, analyses of heat-killed LAB using zebrafish are scarce, except for studies focused on fishery applications [18].

Although the mechanism behind the antioxidant activity of LAB is not fully understood, it has been suggested that LAB may exert antioxidant effects by scavenging reactive oxygen species with its own antioxidant enzymes, chelating metals, and/or modulating the host microbiota [19,20,21]. It has also been proposed that LAB may indirectly enhance antioxidant activity by increasing the levels of antioxidant enzymes in the host animals. The involvement of the Keap1-Nrf2 pathway has been suggested for these indirect antioxidant effects, but this has not been genetically demonstrated using Nrf2-deficient animals. Therefore, this study aims to investigate the antioxidant properties of heat-killed LAB at the animal level using zebrafish. If antioxidant activity is observed, we will verify whether it is mediated through the Keap1-Nrf2 pathway using Nrf2-knockout zebrafish.

## 2. Results

### 2.1. Heat-Killed LAB Showed Antioxidant Activities in Zebrafish Larvae

**Figure 1:**
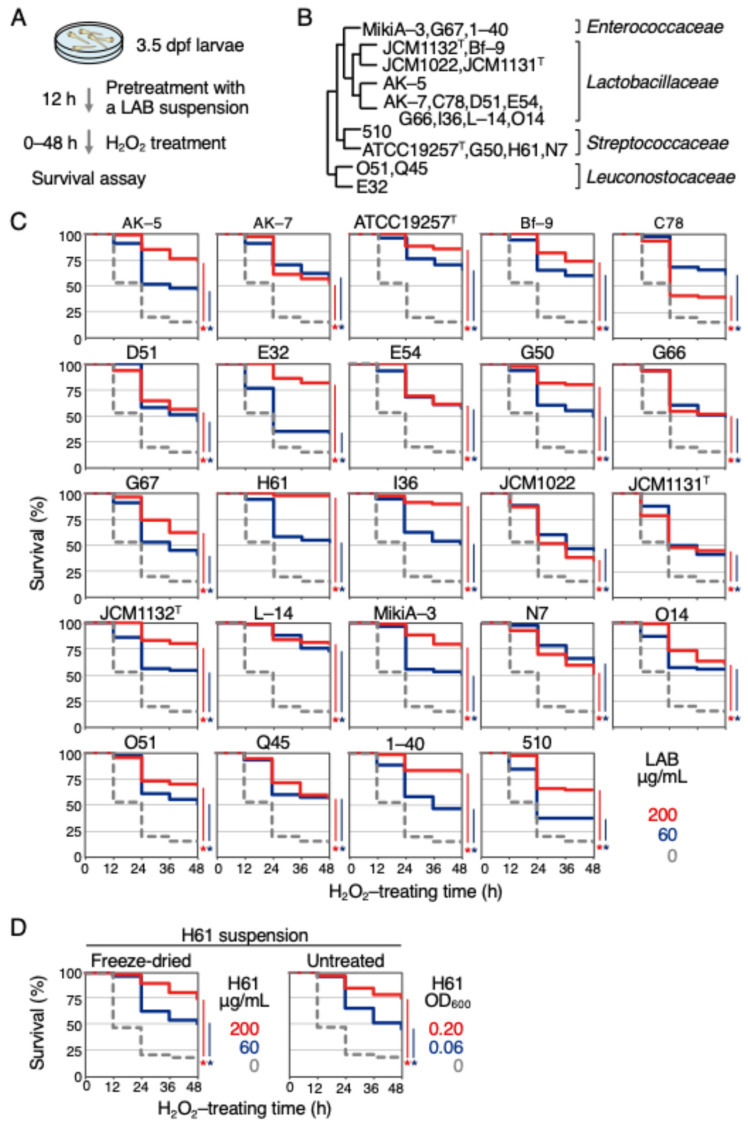
Effects of pretreatment with LAB on the survival rates of zebrafish larvae exposed to H_2_O_2_. (**A**) A schematic diagram of the assay. Twenty 3.5 dpf larvae were placed into a 3 cm dish, pretreated with LAB suspension for 12 h, then exposed to 2.0 mM H_2_O_2_ for 48 h, and the survival rates of the exposed larvae were measured every 12 h. (**B**) Phylogenetic evolutionary tree of LAB strains used in this experiment. ATCC: American Type Culture Collection, JCM: Japan Collection of Microorganisms. (**C**) Antioxidant activity in suspensions of 24 LAB strains. 3.5 dpf larvae were pretreated with suspensions of the indicated LAB strains at concentrations of 0 (dotted gray), 60 (blue), or 200 µg/mL (red). After pretreatment for 12 h, the solution was replaced with 2.0 mM H_2_O_2_, and survival was measured every 12 h for 48 h. Each analysis was performed in duplicate, and the total number of tested larvae was more than 60 in each case (N > 60). (**D**) Comparison of the antioxidant activity of freeze-dried and non-freeze-dried samples. A total of 3.5 dpf larvae were pretreated with suspension of the strain H61 at indicated concentrations. After pretreatment for 12 h, the solution was replaced with 2.0 mM H_2_O_2_, and survival was measured every 12 h for 48 h. Each analysis was performed in quadruple (N > 130). Asterisks indicate *p* values of <0.01, which are considered statistically significant. OD_600_: optical density at 600 nm.

To investigate the antioxidant activities of LAB, a previously established zebrafish assay system was used (Figure 1A) [10,11]. Hydrogen peroxide (H_2_O_2_) was used as the oxidative stressor. Various types of LAB were examined, specifically, 3 strains of *Enterococcaceae* (MikiA-3, 1-40, G67), 13 strains of *Lactobacillaceae* (JCM1132^T^, Bf-9, JCM1022, JCM1131^T^, AK-5, AK-7, C78, D51, E54, G66, I36, L-14, O14), 5 strains of *Streptococcaceae* (510, ATCC19257^T^, G50, H61, N7), and 3 strains of *Leuconostocaceae* (O51, Q45, E32) (Table 1, Figure 1B). All strains were heat-killed and then freeze-dried into powder for use. Exposure of 4 days-post fertilization (dpf) zebrafish larvae to 2.0 mM H_2_O_2_ resulted in 86% mortality within 48 h (Figure 1C, dotted gray).

When larvae were pretreated with LAB solution at concentrations of 60 or 200 μg/mL for 12 h, their survival rates significantly increased for all 24 strains at both concentrations (Figure 1C, blue and red lines, respectively). However, the effectiveness varied among the bacteria. At 200 μg/mL, the most effective was strain H61, which reduced mortality to 3%, followed by strain I36 to 11%, and strain ATCC 19257^T^ to 15%, respectively. The least effective strains were JCM1022 at 65%, C78 at 61%, and JCM 1131^T^ at 55%. These results suggest that LAB possess antioxidant activity of varying effects, regardless of their family or genus, and also imply that the mechanism of antioxidant activity is common among these bacteria. Therefore, to elucidate this mechanism, the antioxidant activity of strain H61, which was shown to have the highest antioxidant activity, was focused on for further analysis. For the subsequent analyses of the strain H61, a suspension of the LAB without freeze-drying was used. As shown in Figure 1D, about 83% of the larvae died after 48 h of H_2_O_2_ exposure, while the mortality rates were reduced to 25% (200 μg/mL) and 49% (60 μg/mL) when the tested larvae were pretreated for 12 h with a suspension of freeze-dried H61. Similarly, the mortality rates were reduced to 25% (0.20 optical density at 600 nm (OD_600_)) and 54% (0.06 OD_600_) when the larvae were pretreated with an H61 suspension without freeze-drying. This result confirmed that either form of H61 suspension, with or without freeze-drying, can be used for analysis (Figure 1D).

### 2.2. H61 Suspension Exhibited Indirect but Not Direct Antioxidant Activity

**Figure 2:**
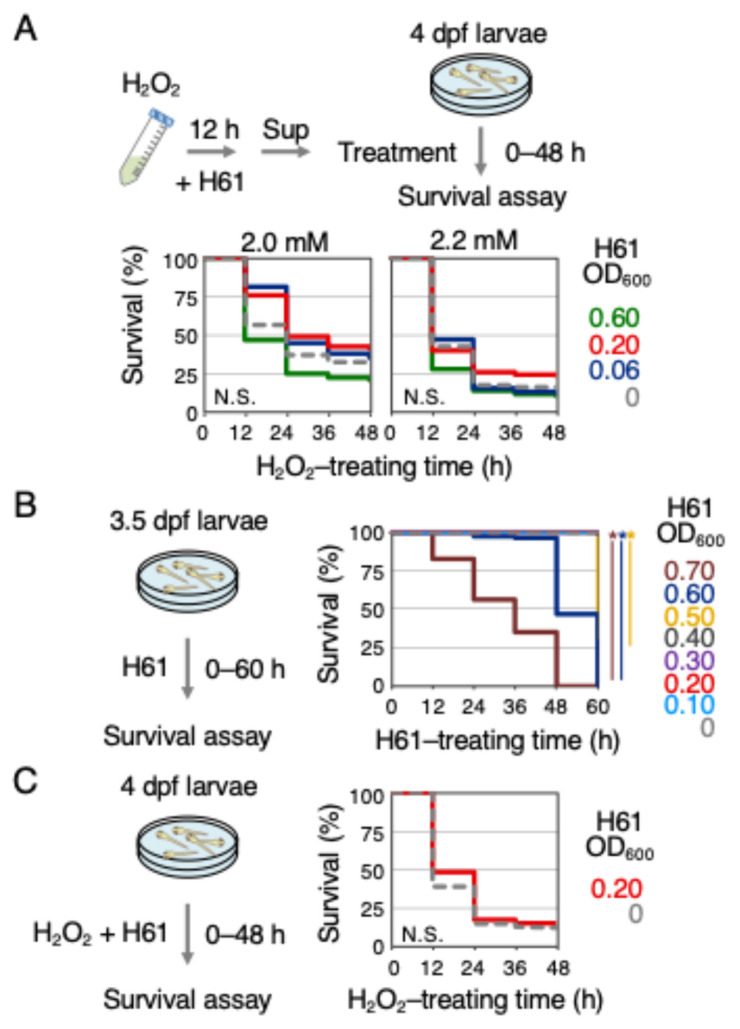
Verification of the direct antioxidant activity of heat-killed H61 suspension in zebrafish larvae. (**A**) Direct antioxidant activity of H61 suspension. H_2_O_2_ solution (2.0 or 2.2 mM) was mixed with the H61 suspension at concentrations of 0 (dotted gray), 0.06 (blue), 0.20 (red), or 0.60 OD_600_ (green) for 12 h in a 50 mL tube, and then H61 was removed from the mixed H_2_O_2_–H61 solution by centrifugation (Sup). Survival rates of larvae exposed to H61-pretreated H_2_O_2_ were measured every 12 h for 48 h. Each analysis was performed in duplicate or more (N > 70). (**B**) Toxic activity of H61 suspension. Here, 4 dpf larvae were treated with the H61 suspension at concentrations of 0.10 to 0.70 OD_600_ for 60 h (light blue, 0.10 OD_600_; red, 0.20; purple, 0.30; dark gray, 0.40; yellow, 0.50; blue, 0.60; brown, 0.70). Survival rates were measured every 12 h. Each analysis was performed in duplicate (N > 70). (**C**) Effect of concurrent treatment with H61 suspension and H_2_O_2_. Here, 4 dpf larvae were treated with a mixture of 2.0 mM H_2_O_2_ and the H61 suspension (red, 0.20 OD_600_; doted gray, 0 OD_600_). Survival rates were measured every 12 h. Each analysis was performed in triplicate (N > 80). Asterisks indicate *p* values of <0.01, which are considered statistically significant.

It is known that live LAB has direct antioxidant activities [20,21]. Despite being heat-killed, the antioxidant activities observed in the experiments might include not only indirect but also direct antioxidant activities. If H61 has direct antioxidant activity, pretreating H_2_O_2_ with H61 should reduce the lethality of H_2_O_2_ for the zebrafish larvae. Therefore, the lethality of H_2_O_2_ pretreated with H61 for 12 h in vitro was compared to that of untreated H_2_O_2_ on 4 dpf larvae (Figure 2A). As a result, 59% and 65% of 4 dpf zebrafish larvae exposed to H_2_O_2_ (2.0 mM) pretreated in vitro with H61 (0.20 and 0.06 OD_600_, respectively) died after 48 h of treatment. These mortality rates were not significantly different from those observed in larvae exposed to H_2_O_2_ alone (69%), suggesting that the H61 treatment had no effect. Considering the possibility that the direct antioxidant activity might be smaller than the indirect activity, the concentration of H61 was increased to 0.60 OD_600_ for analysis, but the same result (79%) was obtained. To be thorough, the effects were also analyzed at a concentration of 2.2 mM H_2_O_2_, but similar results were observed. Briefly, 90%, 77%, 88%, and 84% of larvae exposed to H_2_O_2_ pretreated with H61 (0.60, 0.20, 0.06, and 0 OD_600_, respectively) died after 48 h of treatment. These observations suggest that the heat-killed H61 suspension does not have direct antioxidant activity.

When the concentration was raised to 0.60 OD_600_, there was a tendency for the lethality of H_2_O_2_ to increase, suggesting the possibility of toxicity at high concentrations of the H61 suspension. Therefore, a toxicity test was conducted with varying concentrations of H61 (Figure 2B). When treating 4 dpf larvae with H61 suspensions ranging from 0.10 to 0.70 OD_600_ and examining the survival rate over 60 h, there was no impact up to 0.40 OD_600_ (0.10–0.30 OD_600_, 0% lethal; 0.40 OD_600_, 1% lethal), but lethality appeared at 0.50 OD_600_ (0.50 OD_600_, 68% lethal; 0.60–0.70 OD_600_, 100% lethal). This result suggests that H61 suspensions of 0.40 OD_600_ or less do not exhibit toxicity to zebrafish, at least for a treatment duration of two and a half days. Next, an experiment was conducted where H61 suspension and H_2_O_2_ were treated simultaneously (Figure 2C). It was found that simultaneous addition of H61 suspension, unlike with the pretreatment in Figure 1D, had no impact on the lethality of H_2_O_2_ (86% lethal; control 88%). The result of the simultaneous treatment suggests that it takes time for strain H61 to exhibit antioxidant activity.

### 2.3. Indirect Antioxidant Activity Induced by Strain H61 Was Independent of the Keap1-Nrf2 Pathway

**Figure 3:**
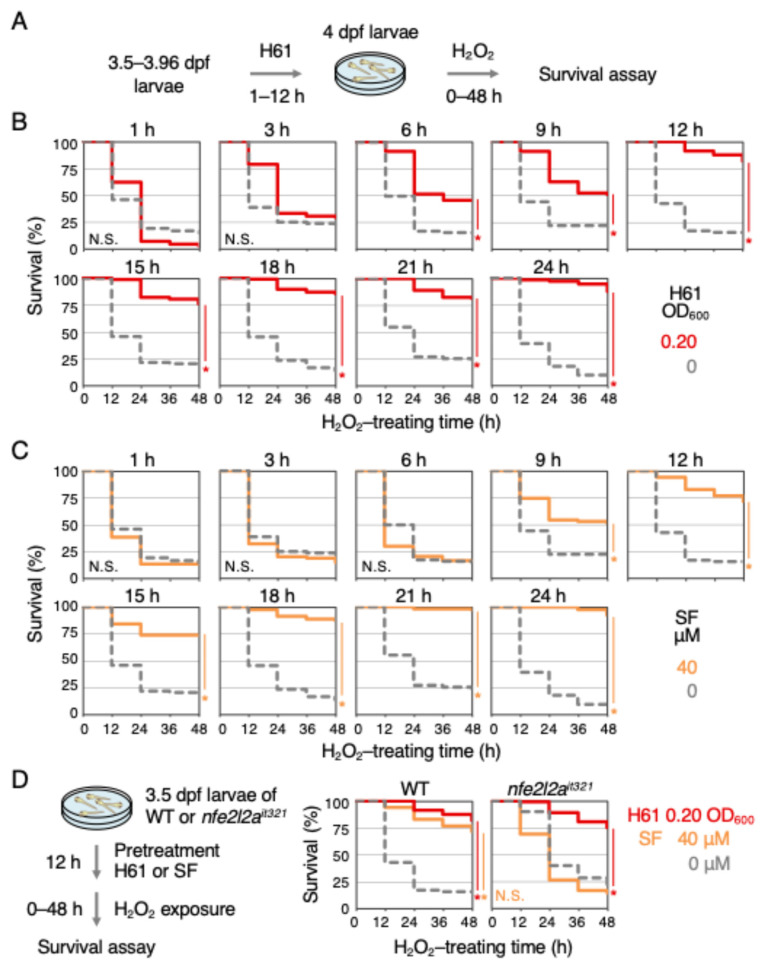
Relationship between the antioxidant activity of heat-killed H61 suspension and the Keap1-Nrf2 pathway (**A**) A schematic diagram of the assay. Twenty zebrafish larvae (3.5–3.96 dpf) were placed into a 3 cm dish, pretreated with 0.20 OD_600_ H61 suspension for 1–24 h, and then exposed to 2.0 mM H_2_O_2_ for 48 h, and the survival rates of the treated larvae were measured every 12 h. (**B**,**C**) Effects of pretreatment time. Larvae were pretreated for the indicated time without (dotted gray) or with 0.20 OD_600_ H61 suspension (red) (**B**) or 40 µM sulforaphane (SF) (orange) (**C**). After pretreatment, the solution was replaced with 2.0 mM H_2_O_2_, and survival was measured every 12 h for 48 h. Each analysis was performed in duplicate or more (N > 65). (**D**) Effect of Nrf2 disruption. The 3.5 dpf larvae of wild-type (WT) or homozygous knockout zebrafish (*nfe2l2a^it321^*) were pretreated without (dotted gray) or with 0.20 OD_600_ H61 suspension (red) or 40 μM sulforaphane (yellow) for 12 h and then exposed to H_2_O_2_ (wild-type, 2.0 mM; *nfe2l2a^it321^*, 1.4 mM), and survival was measured every 12 h for 48 h. Each analysis was performed in duplicate or more (N > 90). Asterisks indicate *p* values of <0.01, which are considered statistically significant.

The pretreatment time required for strain H61 to exhibit its antioxidant activity was next examined (Figure 3A). The start time of H61 pretreatment was gradually delayed, starting at 3.5 dpf with a 12 h pretreatment and reducing it to as much as 3.96 dpf with a 1 h pretreatment. At 4 dpf, H61-pretreated larvae were exposed to H_2_O_2_, and their survival was analyzed (Figure 3B). As a result, no antioxidant activity was observed with 1 h or 3 h pretreatments, but a significant increase in antioxidant activity was observed with pretreatment of 6 h or more (6 h 54% lethal, control 85%; 9 h 49%, control 79%; 12 h 18%, control 85%). This suggests that more than 6 h is required for H61 components to enter the fish’s body and exhibit antioxidant activity. The antioxidant activity of Nrf2 activators, such as sulforaphane and auranofin, have also been shown to require pretreatment time [8,22].

Because the pretreatment time required for sulforaphane to exhibit its indirect antioxidant activity had not previously investigated, a similar analysis was conducted for sulforaphane as was performed for the H61 suspension. While no antioxidant activity was observed with 6 h or less of sulforaphane pretreatment, it was observed with 9 h or more (Figure 3C) (6 h 84% lethal; 9 h 49%; 12 h 28%). This result shows that Nrf2 activation by sulforaphane and the subsequent antioxidant activity induced by Nrf2 requires more than 9 h. Although the required pretreatment time is slightly different, it became clear that it is similar to that of the H61 suspension. Therefore, Nrf2-knockout zebrafish *nfe2l2a^it321^* were next used to examine whether the antioxidant activity of strain H61 was dependent on the Keap1-Nrf2 pathway (Figure 3D) [11]. The concentration of H_2_O_2_ was set at 1.4 mM because Nrf2-knockout larvae had weaker defenses against H_2_O_2_ compared to wild-type larvae. The results showed that the antioxidant activity of sulforaphane was lost in the Nrf2-knockout mutants (48 h: WT 28% lethal, control 85%; Nrf2-knockout 86%, control 79%), while that of strain H61 was almost unaffected (WT 18% lethal; Nrf2-knockout 25%). This suggests that the antioxidant activity of H61 is Nrf2-independent and does not involve the Keap1-Nrf2 pathway.

### 2.4. Strain H61 Did Not Exhibit Antioxidant Activity against Arsenite

**Figure 4:**
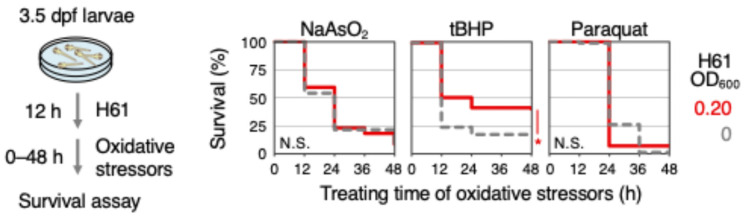
Effects of pretreatment with heat-killed H61 suspension on the toxicities of various oxidative stressors. The 3.5 dpf larvae were pretreated without (dotted gray) or with 0.20 OD_600_ H61 suspension (red) for 12 h. They were then exposed to 1.0 mM NaAsO_2_, 1.25 mM tBHP, or 5.5 mM paraquat, and survival was measured every 12 h for 48 h. Each analysis was performed in duplicate (N > 80). Asterisks indicate *p* values of <0.01, which are considered statistically significant.

It has been previously shown that sulforaphane exhibits Nrf2-dependent antioxidant activity against another oxidative stressor, sodium arsenite (NaAsO_2_) [9]. The antioxidant activity of strain H61 found in this study is Nrf2-independent and acts through a different mechanism other than sulforaphane, suggesting that its activity against NaAsO_2_ may differ from that of Nrf2 activators like sulforaphane. Therefore, the effect of H61 pretreatment on NaAsO_2_ toxicity was analyzed (Figure 4). The results showed that treatment of 1 mM NaAsO_2_ killed about 92% of the larvae within 48 h, and pretreatment with strain H61 did not reduce this lethality (92%). Similar analyses were conducted on *tert*-butyl hydroperoxide (tBHP) and paraquat, other oxidative stressors used in a previously study [8] (Figure 4). Exposure to 1.25 mM tBHP resulted in about 82% larvae mortality within 48 h, while the mortality rate was reduced to 59% with H61 pretreatment. Exposure to 5.5 mM paraquat resulted in total mortality of 100% of larvae after 48 h, while pretreatment with strain H61 resulted in mortality of about 93% of larvae. In summary, H61 pretreatment mitigated the toxicity of H_2_O_2_ and tBHP but did not alleviate the toxicity of NaAsO_2_ and paraquat. These findings indicate that the antioxidant activity of strain H61 is not universally applicable to all oxidative stressors.

## 3. Discussion

In this study, using a zebrafish larvae assay system, we found that all 24 strains of heat-killed LAB showed antioxidant activity that alleviated H_2_O_2_ toxicity. The results suggest that zebrafish larvae are an excellent model for evaluating the antioxidant activity of heat-killed LAB and further imply that LAB fundamentally possess the ability to enhance cellular defense against oxidative stress. In fact, LAB are suggested to possess indirect antioxidant activity by activating the antioxidant enzymes in treated cells and animals [20]. For example, *Lactiplantibacillus plantarum* Y44 and *Lacticaseibacillus casei* Shirota have been shown to enhance the antioxidant capacity of Caco-2 human colon cancer cells by increasing the activities of catalase and glutathione peroxidase [23,24]. It has also been shown that in animals such as rats, pigs, chickens, and zebrafish, oral administration of *Lacticaseibacillus casei* Zhang, *Limosilactobacillus fermentum*, *Levilactobacillus brevis* 23017, and *Lactiplantibacillus plantarum*, respectively, increases the activity of antioxidant enzymes such as superoxide dismutase, glutathione peroxidase, and catalase, thereby enhancing resistance to oxidative stress [25,26,27,28]. *Streptococcus salivarius*, a member of the *Streptococcaceae* family, to which strain H61 belongs, has also been reported to enhance the antioxidant ability of HepG2 human liver cancer cells and MCF-7 human breast cancer cells [29].

Among the 24 strains of LAB, strain H61, belonging to the *Streptococcaceae* family, was found to exhibit the strongest activity. H61 has been widely used in Japan for the production of fermented dairy products over the past 60 years. It has been previously found that oral administration of heat-killed H61 exhibits anti-aging effects in aged senescence-accelerated mice [30] and has preventive effects against age-related loss in middle-aged mice [31]. Effects on promoting human health have also been observed. For example, oral intake of heat-killed H61 has been shown to improve some skin properties [32] and increase serum iron levels [33]. The antioxidant properties of strain H61 identified in this paper may contribute to the basis of these beneficial functions for human and animal health.

It is known that LAB can activate various signaling pathways, such as Keap1-Nrf2, silent information regulator 1 (SIRT1), nuclear factor kappa-light-chain-enhancer of activated B cells (NFκB), mitogen-activated protein kinase (MAPK), and protein kinase C, which are responsible for the response to oxidative stress in the host cells and in animals [20,21]. Among these, the Keap1-Nrf2 pathway is the most promising since its role in the cytoprotection by *Lacticaseibacillus rhamnosus* GG has been shown in the intestine using Nrf2-knockout mice [34]. However, the current analysis showed that the antioxidant activity of H61 was independent of Nrf2, suggesting the involvement of pathways other than Keap1-Nrf2, possibly including unknown pathways. The antioxidant activity of LAB identified by Jones et al. [34] is thought to differ significantly from the antioxidant activity observed in this study due to the following three reasons: (1) their evaluation was based on the ability to reduce mouse mortality, weight loss, and colon cell death; (2) they used γ-irradiation as an oxidative stressor; and (3) they used live LAB. The difference in Nrf2 dependence further supports this distinction.

Although no specific pathways can be identified at this point, the following points are worthy of consideration: (1) because it requires less pretreatment time than the Keap1-Nrf2 pathway, the pathway may not involve gene expression of antioxidant proteins, and (2) it does not exhibit effects against paraquat or arsenite toxicity, suggesting that it may not activate enzymes that scavenge radical reactive oxygen species. On the other hand, from the perspective of LAB, because all tested LAB strains exhibited antioxidant activity to varying degrees, the LAB components responsible for antioxidant activity may be common to all strains. In this case, components such as exopolysaccharides may promote indirect antioxidant activities [35]. Clarifying the mechanism by which LAB exhibit antioxidant activity in zebrafish remains a question for future research to answer. Preliminary analysis has not identified any antioxidant enzymes whose expression is induced by H61 treatment, and it is not expected that this is a mechanism for gene induction, as seen in the Keap1-Nrf2 pathway. A proposed strategy is to identify the components responsible for the antioxidant activity of H61, determine which zebrafish proteins interact with these components, and provide genetic evidence for this through gene-knockout studies.

In conclusion, we have discovered a novel indirect antioxidant activity in heat-killed LAB that is Nrf2-independent. Previously, four dietary phytochemicals—sulforaphane, curcumin, quercetin, and diary trisulfide—were identified as mitigating H_2_O_2_ toxicity in an assay system using zebrafish larvae [10], all of which were Nrf2-dependent. In contrast, the activity exhibited by LAB is noteworthy because it represents the first Nrf2-independent antioxidant activity.

## 4. Materials and Methods

### 4.1. Zebrafish and Chemicals

In this study, AB (wild-type) and Nrf2-knockout (*nfe2l2a^it321^*) [11] zebrafish larvae were used. The knockout lines were maintained by PCR-based genotyping as described previously [11]. Embryos were obtained by natural mating. H_2_O_2_ and NaAsO_2_ were purchased from FUJIFILM Wako (Osaka, Japan), tBHP and paraquat from Tokyo Chemical Industry (Tokyo, Japan), and sulforaphane from LKT Laboratories (St. Paul, MN, USA). H_2_O_2_, NaAsO_2_, tBHP, and paraquat were dissolved in MilliQ water (Merck-Millipore, Billerica, MA, USA), and sulforaphane in ethanol, for the stock solution. They were diluted to final concentrations with E3+ medium (5 mM NaCl, 0.17 mM KCl, 0.33 mM CaCl_2_, 0.33 mM MgSO_4_, and 0.1 μg/mL methylene blue).

### 4.2. LAB Preparation

LAB strains used in this study are listed in Table 1. They were cultured in de Man, Rogosa, and Sharpe (MRS) broth (Becton Dickinson, Cockeysville, MD, USA) by subculturing 0.5% inocula for 18–24 h at 30 °C or 37 °C for their optimum temperature, as previously described [36] with slight modification. The bacterial cells were harvested and washed twice with 0.85% NaCl, then resuspended in the MilliQ water, followed by heat-killing at 121 °C for 15 min. Samples were freeze-dried and resuspended in 1 mL of E3+ medium at a final concentration of 1 mg/mL. For H61 suspension without freeze-drying, bacteria concentration was shown as OD_600_. Stock suspensions of LAB were stored at –80 °C, and when used, they were diluted to final concentrations with E3+ medium.

### 4.3. Survival Assays

Survival assays were performed as previously described [9] with slight modification (Figure 1A). Briefly, larvae (3.5 dpf, 20 larvae per well in a standard condition) were placed in a 35 mm dish with 5 mL of LAB suspension (E3+ medium containing each LAB) for 12 h. At 4 dpf, the LAB suspension was replaced with oxidative-stressor solution (E3+ medium containing each oxidative stressor). The survival of larvae was observed every 12 h for 48 h after starting the oxidative-stressor treatment. Each analysis was performed in duplicate or more, and the experiments were repeated multiple times to confirm reproducibility. Larvae were not fed during the experiment. All animal experiments were performed in accordance with the animal protocols approved by the Animal Experiment Committee of the University of Tsukuba (Approval number: 22-444; approval date: 1 June 2022). All methods were carried out in accordance with the Regulation for Animal Experiments in our university and the Fundamental Guideline for Proper Conduct of Animal Experiment and Related Activities in Academic Research Institutions under the jurisdiction of the Ministry of Education, Culture, Sports, Science and Technology, Japan.

### 4.4. Statistical Analysis

All survival data were calculated using the Kaplan–Meier method and analyzed with the log-rank test; *p* values < 0.01 were considered statistically significant. All statistical analyses were performed using EZR (version 4.3.2), which is a graphical user interface for R (The R Foundation for Statistical Computing, Vienna, Austria). More precisely, it is a modified version of R commander designed to add statistical functions frequently used in biostatistics.

## Figures and Tables

**Table 1:** LAB used in this study.

Strains	Species	Origin
AK-5	*Limosilactobacillus fermentum*	Fermented vegetable
AK-7	*Lactiplantibacillus plantarum*	Fermented vegetable
ATCC 19257^T^	*Lactococcus cremoris*	Raw milk
Bf-9	*Lactobacillus helveticus*	Unknown
C78	*Latilactobacillus curvatus*	Fermented vegetable
D51	*Latilactobacillus sakei*	Fermented vegetable
E32	*Weisella cibaria*	Fermented vegetable
E54	*Lactiplantibacillus paraplantarum*	Fermented vegetable
G50	*Lactococcus lactis* subsp. *lactis*	Napiergrass
G66	*Pediococcus pentosaceus*	Pasteurized milk
G67	*Enterococcus feacium*	Pasteurized milk
H61	*Lactococcus cremoris*	Cheese starter
I36	*Levilactobacillus brevis*	Fermented vegetable
JCM 1022	*Lactobacillus johnsonii*	Human feces
JCM 1131^T^	*Lactobacillus gasseri*	Human feces
JCM 1132^T^	*Lactobacillus acidophilus*	Human feces
L-14	*Lacticaseibacillus casei*	Cheese
MikiA-3	*Enterococcus feacalis*	Fermented rice milk
N7	*Lactococcus lactis* subsp. *lactis* biovar diacetylactis	Cheese starter
O14	*Lacticaseibacillus paracasei*	Homemade kefir
O51	*Leuconostoc mesenteroides*	Fermented vegetable
Q45	*Leuconostoc citreum*	Fermented vegetable
1-40	*Enterococcus munditii*	Fermented vegetable
510	*Streptococcus thermophilus*	Cow milk

Abbreviations: ATCC, American Type Culture Collection; JCM, Japan Collection of Microorganisms.

## Data Availability

The raw data supporting the conclusions of this article will be made available by the authors on request.
